# Continuous Positive Airway Pressure (CPAP) for severe pneumonia in low- and middle-income countries: A systematic review of contextual factors

**DOI:** 10.7189/jogh.12.10012

**Published:** 2022-10-30

**Authors:** Chris Wilkes, Rami Subhi, Hamish R Graham, Trevor Duke, the ARI Review group, Steve Graham, Amy Gray, Amanda Gwee, Claire von Mollendorf, Kim Mulholland, Fiona Russell, Maeve Hume-Nixon, Saniya Kazi, Priya Kevat, Eleanor Neal, Cattram Nguyen, Alicia Quach, Rita Reyburn, Kathleen Ryan, Patrick Walker, Poh Chua, Yasir Bin Nisar, Jonathon Simon, Wilson Were

**Affiliations:** 1 Murdoch Children’s Research Institution, Royal Children’s Hospital, Parkville, Victoria, Australia; 2 Department of Paediatrics, University of Melbourne, Parkville, Victoria, Australia

## Abstract

**Background:**

Continuous positive airway pressure (CPAP) may have a role in reducing the high mortality in children less than 5 years with World Health Organization (WHO) severe pneumonia. More evidence is needed to understand important contextual factors that impact on implementation, effectiveness, and safety in low resource settings.

**Methods:**

We conducted a systematic review of Medline, Embase and Pubmed (January 2000 to August 2020) with terms of “pneumonia”, “CPAP” and “child”. We included studies that provided original clinical or non-clinical data on the use of CPAP in children (28 days-4 years) with pneumonia in low- or middle-income countries. We used standardised tools to assess study quality, and grade levels of evidence for clinical conclusions. Results are presented as a narrative synthesis describing context, intervention, and population alongside outcome data.

**Results:**

Of 902 identified unique references, 23 articles met inclusion criteria, including 6 randomised controlled trials, one cluster cross over trial, 12 observational studies, 3 case reports and 1 cost-effectiveness analysis. There was significant heterogeneity in patient population, with wide range in mortality among participants in different studies (0%-55%). Reporting of contextual factors, including staffing, costs, and details of supportive care was patchy and non-standardised. Current evidence suggests that CPAP has a role in the management of infants with bronchiolitis and as escalation therapy for children with pneumonia failing standard-flow oxygen therapy. However, CPAP must be implemented with appropriate staffing (including doctor oversight), intensive monitoring and supportive care, and technician and infrastructure capacity. We provide practical guidance and recommendations based on available evidence and published expert opinion, for the adoption of CPAP into routine care in low resource settings and for reporting of future CPAP studies.

**Conclusions:**

CPAP is a safe intervention in settings that can provide intensive monitoring and supportive care, and the strongest evidence for a benefit of CPAP is in infants (aged less than 1 year) with bronchiolitis. The available published evidence and clinical experience can be used to help facilities assess appropriateness of implementing CPAP, guide health workers in refining selection of patients most likely to benefit from it, and provide a framework for components of safe and effective CPAP therapy.

**Protocol registration:**

PROSPERO registration: CRD42020210597.

Every year 900 000 children under the age of 5 die of pneumonia [1]. Preventative and curative measures in the community and within health facilities are effective in reducing the burden of pneumonia, and their coverage needs to be increased [2]. However, mortality for children with respiratory failure, not responding to standard treatment, continues to be high, and there may be a role of continuous positive airway pressure (CPAP) as an additional therapy.

CPAP has an established role for the management of respiratory distress syndrome in preterm babies [3], and is commonly used in the treatment of bronchiolitis in older infants [4]. Multiple studies of bubble CPAP in low resource settings have documented mortality reductions in special care baby units [5,6], but highlighted the resource implications, and importance of broader contextual factors in implementation [7].

Conversely, the literature has been less clear on the role of CPAP for pneumonia, particularly in district and secondary hospitals in low- and middle-income countries (LMICs), where collectively, the greatest number of cases are managed. Two systematic reviews and meta-analyses have reported on the effectiveness of CPAP for children with severe pneumonia in LMICs [8,9] finding mixed results on mortality and trend towards higher adverse events rates in CPAP compared to standard low-flow oxygen. Both reviews noted the heterogeneity in populations and context and the likely implications these factors have on safety and efficacy. And both reviews recommended further randomised controlled trials, with Sessions suggesting that CPAP implementation in resource-limited settings be restricted to well-staffed intensive care or high dependency settings until further data are available [8].

This review aimed to increase understanding of the patient selection and contextual factors that influence effectiveness and safety of CPAP for children with severe pneumonia. We also aimed to review other factors around implementation including feasibility, sustainability, and acceptability in resource-limited settings, in order to inform global recommendations and highlight gaps in the literature for future implementation research.

## METHODS

We conducted a systematic search of medical databases Medline, Embase, and PubMed for all relevant articles between January 1, 2000 to August 19, 2020. We mapped search terms to medical subject headings where possible, using Boolean operators to combine searches into our final systematic search query. We used synonyms of “pneumonia”, “CPAP”, and “child” to target our search strategy, with oversight from an experienced Health Service Librarian to ensure all relevant papers were identified. We also searched reference lists of all included references for eligible studies. The specific search terms used for our Medline search and further details of the search strategy, information sources and data collection processes are included in Appendix S1-S2 in the **Online Supplementary Document**.

### Assessment of study eligibility

Two reviewers (CW and RS) independently screened the titles and abstracts of all returned studies. We obtained full text for studies that were screened in by either reviewer, and the same two reviewers independently assessed them for inclusion. We included studies involving CPAP for children (aged 28 days to 4 years) with World Health Organization (WHO) pneumonia/severe pneumonia conducted in a LMIC. We focussed on more recent studies (published after the year 2000) to maximise contextual relevance and excluded studies that focussed on a neonatal population (Table S1 in the **Online Supplementary Document**). We did not exclude studies of CPAP in known viral aetiology of lower respiratory infection if these children fulfilled the WHO definition of pneumonia (cough or difficulty breathing with tachypnoea or chest wall indrawing). We resolved disagreements by discussion and, where appropriate, review by a third reviewer (HG).

### Data management, extraction, and synthesis

We used a standardised data extraction form to extract data relevant to our review. Two reviewers (CW and RS) independently extracted data from each eligible study and entered data into an Excel spreadsheet (Microsoft, Redmond, US). We resolved disagreements by discussion and contacted study authors where appropriate to resolve any uncertainties. We extracted data on the study population (age, sex, comorbidities), CPAP details (type, humidification, oxygen source, air/oxygen mix, etc.), clinical guidelines, monitoring and supportive care (starting pressure and FiO_2_, decision-making algorithm, fluids/feeding, etc.), health service characteristics (level of care, staffing, etc.), and outcomes (mortality, adverse events, treatment failure, etc.) (detail in Appendix S1 in the **Online Supplementary Document**).

We used tables and narrative synthesis to describe context, intervention, and population and triangulate this with the reported outcome and safety data (using the effect measures reported in each original study) We did not conduct a meta-analysis of CPAP outcomes, as this has been reported previously [8] and the focus of this study was to understand contextual and population factors. Heterogeneity in study outcomes, populations and contextual factors reported in included studies prevented quantitative synthesis of potential associations. Rather, we triangulated context, intervention, and population data with outcome data to enable qualitative synthesis. We graded levels of evidence for key findings using Oxford Levels of Evidence framework, where 1 is the highest level of evidence (typically systematic review of trials) and 5 is the lowest level of evidence (typically mechanism-based reasoning) [10].

### Assessment of study quality and risk of bias

We assessed the quality and risk of bias of all included studies by using the Effective Public Health Practice Project (EPHPP) Quality Assessment Tool [11]. Using this tool, two reviewers (CW and RS) independently rated study quality as strong, moderate or weak with respect to selection bias, study design, confounders, blinding, data collection method, withdrawals and dropouts, and a global rating. Where disagreements occurred, a third reviewer (HG) carried out a final assessment. Evidence for recommendations and conclusions was graded using the Oxford Centre for Evidence Based Medicine levels of evidence [10].

## RESULTS

**Figure 1:**
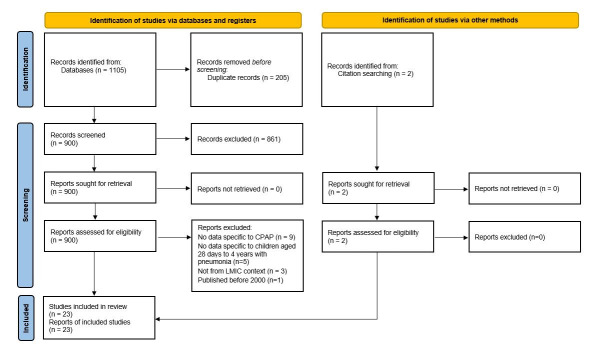
Preferred Reporting Items for Systematic Reviews and Meta-Analyses (PRISMA) flow diagram. CPAP - continuous positive airway pressure, LMIC - low- or middle-income country.

Our search identified 1105 articles, with 2 additional articles identified on review of references. After duplicates were removed 902 references were screened, including full text review of 41 publications, of which 18 were excluded (**Figure 1**): 9 because it was not possible to extract data specific to patients who received CPAP [12-24], 4 because it was not possible to extract data specific to post-neonatal patients [25-28], 3 because it was not possible to extract data specific to low and middle income countries [4,29,30], 1 because it was a pooled analysis which included studies in which most patients did not have pneumonia [31], and 1 because it was published prior to the year 2000 [32].

We included 23 studies in qualitative synthesis, including six individually randomised controlled trials (RCTs) [33-38], one cluster open-label crossover trial [39], twelve observational studies [40-51], one cost-effectiveness analysis [52], and three case reports (**Table 1**) [53-55]. Four studies reported on CPAP sustainability [51], health workforce implications [48], parental attitudes [49], or cost-effectiveness [52], but did not provide clinical data.

**Table 1:** Characteristics of included studies

Paper	Study setting and Context	Population	Intervention	Comparison	Primary outcome	Key findings	Adverse Events
**Bronchiolitis**							
Cesar 2018 [33] RCT (n = 62)	São Paulo, Brazil, urban tertiary hospital, PICU. Nurse: patient ratio not reported, review frequency not reported, doctor presence not reported, CPAP training details not reported, CXR not reported, NGT use not reported	<9 m of age with clinical bronchiolitis, median age 2.4 mo in CPAP arm, 3.4 mo in high flow arm, mortality not reported	CPAP	High flow nasal cannula (HFNC)	Treatment failure defined as progression to worsening respiratory failure requiring escalation of support	Treatment failures: 10 (35.7%) CPAP vs 13 (38.2%) HFNC, *P* = 0.952. No differences in length of hospital stay, time in PICU, or duration of respiratory support needed.	Not reported
Lal 2018 [35], RCT (n = 72)	Delhi, India, urban tertiary hospital, paediatric ward. Nurse: patient ratio not reported, review frequency: at 0 and 1 h, doctor presence not reported, CPAP training details not reported, CXR available, NGT use not reported	Clinical diagnosis of bronchiolitis (respiratory rate ≥50/min, infant aged 1 mo to 1 y, with wheezing and hyperinflated lungs). Excluded: infants in imminent need of ventilator support, Median age 4 mo (CPAP), 4.7 mo (standard). No patients died	Bubble CPAP	Standard Care	Change in respiratory rate after the first hour of treatment	Change in respiratory rate> = 10: 44% (n = 14) CPAP vs 14% (n = 5) standard, *P* = 0.008. Change in respiratory rate 8.0 vs 5.1 *P* = 0.02. Change in Silverman-Anderson score 0.78 vs 0.39 *P* = 0.029. Change in modified PSNZ severity score 2.5 vs 1.08 *P* = 0.012	None (including nasal mucosal damage, mucosal excoriation, scarring, pressure necrosis, pneumothorax or decreased cardiac output)
Sarkar 2018 [37], randomised pilot study (n = 31)	Kolkata, India, urban tertiary hospital, PICU. Nurse: patient ratio not specifically reported, but “intensively observed by a trained nurse”, review frequency: not specifically reported, but endpoint assessments done at 2, 6, 12, 24, 36, and 48 h. Doctor presence not reported, nurses trained in CPAP use, CXR in all subjects at enrolment, arterial line and serial arterial blood gas monitoring in all patients, NGT placed for enteral feeding	28 d-12 mo, severe bronchiolitis, and peripheral SpO_2_<92% in air and/or respiratory distress assessment Index (RDAI)≥11. Excluded: need for intubation (GCS<11, arterial pH<7.25, arterial Paco_2_>55 mm Hg), impaired cough or gag, upper-airway obstruction, face/abdo surgery, hemodynamic instability, cyanotic congenital heart disease or pulmonary vascular anomalies. Median age 3.4 mo (2.8 mo CPAP, 4 mo high flow). No patients died	CPAP via mechanical ventilator	High flow nasal cannula (HFNC)	Need for mechanical ventilation, defined by improvement in heart rate, respiratory rate, respiratory distress, SpO_2_, Paco_2_, Pao_2_ and COMFORT Score	1 patient in each group (6.5%) required intubation. Mean duration of CPAP and HFNC, and of PICU length of stay, were comparable. Improvements in all other end-points tested were comparable for both groups.	Air leaks and skin sores more frequent in CPAP. Nasal injury: CPAP 75% (n = 12) vs HFNC 26.7% (n = 4). No major adverse events (including cardiac arrest, pneumothorax, or safety system failures). No differences in gastric distension or eye irritation
**General respiratory distress**							
Anitha 2016 [40], observational (n = 214)	Tamil Nadu, India, urban tertiary hospital, PICU. Nurse: patient ratio not reported, review frequency: hourly and after every intervention, doctor presence not reported, details of CPAP training not reported, CXR availability not reported, NGT use if abdominal distension	1m-12y clinically requiring CPAP. Excluded: unmaintainable airway, decreased consciousness. Pneumonia 49%, bronchiolitis 31%, 79% of children were <12 mo, mortality 3.27%	CPAP via flow inflating device	None	Decrease in respiratory rate, work of breathing, heart rate, and improvement in sensorium	89.7% of patients on CPAP had treatment success. 98% of clinical bronchiolitis had treatment success	7.8% of patients had CPAP-related adverse event (including pressure sores, abdominal distension, mucosal dryness), 14.5% of pneumonia cases required intubation, of which 75% had septic shock, 25% of patients with comorbidities required intubation
Bjorkland 2018 [41], observational (n = 83)	Gulu, Uganda, regional hospital, paediatric ward. Nurse: patient ratio not reported, review frequency: at 0, 2, then 6 hourly, doctor presence not reported, CPAP training: dedicated team of six Ugandan nurses received interactive training. All nurses demonstrated CPAP assembly and troubleshooting, CXR available, NGT use if abdominal distension	1m-5y modified Tal score >3 or SpO_2_<92%. Excluded: Pneumothorax, congenital heart or lung disease, facial injury/deformity/trauma, epistaxis, recent abdominal surgery, abdominal distension, agonal respirations, GCS<4 or probable imminent death. Median age 15.6 mo, mortality 9.6%	Bubble CPAP	None	Complications and mortality	Improved modified Tal and severity of illness scores, respiratory rate and O_2_ saturations after 2 h of CPAP (*P* < 0.001). Compared to pre-study population, low odds of severe illness after 2 h CPAP (aOR = 0.19, *P* = 0.02)	6% (n = 5) had mild nasal irritation or abdominal distension. No significant complications (including nasal tissue erosion, severe epistaxis, moderate pneumothorax, unrelieved abdominal distension, aspiration, peritonitis, device breakage)
Machen 2015 [45], observational, (n = 79)	Blantyre, Malawi, urban tertiary hospital. Nurse: patient ratio not reported, review frequency: at start, at 1 h, then twice daily, doctor presence not reported, CPAP training details not reported, CXR availability not reported, NGT use not reported	Weight ≤10 kg, respiratory distress, breathing spontaneously and treating doctor deemed bCPAP appropriate. Excluded: upper airway deformity, diaphragmatic hernia, severe cardiac instability, severe birth asphyxia, not considered neurologically viable. Pneumonia 27%, bronchiolitis 53%, PJP 18.9%, median age 3.7 mo,mortality 29%	Bubble CPAP	None	Mortality rate, length of stay, diagnosis and time on bCPAP. Change in respiratory index of severity in children (RISC) score from 0 to 24 h	71% (n = 56) survived to discharge, 80% (n = 45) of survivors had a lower RISC score after 24 h on CPAP, Doctors felt that CPAP was useful and led to a change in clinical practice, most in bronchiolitis, least in sepsis.	Not reported
Wilson 2013 [38] RCT (n = 70)	4 districts, Ghana, 4 rural hospitals. Nurse: patient ratio not reported, review frequency: at study entry and every 20 min for a period of 2 h, doctor presence not reported, CPAP training: Intensive 4-h didactic and hands-on training session led by 2 experienced neonatal intensive care unit nurses and a paediatric critical care doctor USA. 1 d a week in-service training for 1 mo. Nurses observed successfully placing CPAP on at least 1 paediatric patient. CXR availability not reported. NGT use not reported	3 mo-5 y of age with age-specific fast breathing and at least one of the following: subcostal, intercostal, or supraclavicular retractions or nasal flaring. Excluded: facial skin trauma, inability to protect airway, uncontrollable emesis, poor respiratory effort, respiratory failure needing support, cardiac disease, pneumothorax. 50% malaria positive, median age 13 mo (immediate), 14 mo (delayed), mortality 4.3% (all deaths attributed to severe malaria)	Bubble CPAP	Delayed CPAP, initiated after 1 h	Change in respiratory rate over 1 h	Reduced mean respiratory rate 16 breaths/min (66 to 50, 24% decrease) in immediate CPAP over first hour vs no change (61-60, 2% decrease) in delayed CPAP group, *P* < 0.001, Heart rates did not differ between the two groups	No complications noted
Wilson 2017 [39] cluster crossover trial (n = 2200)	Mampong and Kintampo, Ghana, district and municipal hospital emergency. Nurse: patient ratio not reported, review frequency: 0h, 4 h, 8 h, 12 h, and 24 h, doctor presence: twice daily rounds. 2-4 doctors per hospital. 24/7 enrolment. CPAP training: CPAP applied and managed by existing hospital nurses, trained by study investigators. 6 moly refresher training. CXR and other imaging not available, NGT use not reported	1 mo-5 y, age-specific fast breathing and use of accessory muscles or nasal flaring. Excluded: facial skin breakdown/ trauma, inability to protect airway, persistent emesis, low consciousness, respiratory failure needing support, pneumothorax, asthma, upper airway obstruction, cardiovascular instability. Median age 16.7 mo in CPAP arm, 18.3 mo in control arm, mortality 3.2%	Bubble CPAP	Standard care, including low flow oxygen	Mortality 2 weeks after enrolment	2.5% died in CPAP group, 1.6% died in standard care group. Significant decrease in mortality with CPAP in children under 1 RR = 0 · 40, *P* = · 010). No significant difference in mortality in children aged 1 to 5 y. However, age was not a significant modifier of intervention effect on multivariate analysis. Subgroup analyses by malaria status, SpO_2_<90% or 92%, showed no significant difference in mortality at 2 weeks between CPAP and control groups.	Total: 3% (n = 28) CPAP vs 2% (n = 24) standard care. Vomiting: 2% (n = 20), CPAP vs 2% (n = 21) standard, nasal trauma: 0.5% (n = 5), CPAP vs 0.1% (n = 1) standard. No pneumothorax or other serious adverse events
**Hypoxaemic / high-risk pneumonia**							
Chisti 2015 [34] RCT (n = 225)	Dhaka, Bangladesh, urban tertiary hospital, PICU. Nurse: patient ratio not reported, review frequency: hourly including SpO_2_, doctor presence not reported, CPAP training: nurses CPAP and PICU trained, CXR available, nasogastric feeding, or intravenous fluids if the child had very severe respiratory distress	<5 y, WHO severe pneumonia and SpO_2_<90% in room air. Excluded: Congenital heart disease, asthma, upper-airway obstruction, infants <40 weeks corrected gestational age, children with treatment failure at presentation. Median age 7 mo, mortality 10%	Bubble CPAP	High flow nasal cannula (HFNC) and low flow oxygen (LFNC)	Treatment failure defined as at least 2 of severe hypoxaemia, severe respiratory distress, CO2 > 60, pH<7.2 OR mechanical ventilation, death, leave against medical advice	Treatment failure: 6% CPAP vs 13% HFNC vs 24% LFNC CPAP vs LFNC relative risk (RR) = 0.27, 99.7% CI = 0.07-0.99; *P* = 0.0026 CPAP vs HFNC RR = 0.50, 99.7% CI = 0.11-2.29; *P* = 0.175. Mortality: 4% CPAP vs 13% HFNC vs 15% LFNC, CPAP vs LFNC RR = 0.25, 95% CI = 0.07-0.89; *P* = 0.022	Heart failure 16 (20%), hyponatraemia 7 (9%), hypernatraemia 13 (17%), seizures 4 (5%), nosocomial infection 4 (5%), abdominal distension 1 (1%) – all similar to LFNC and HFNC arms. CPAP technical problem 26 (21%) requiring action
Chisti 2018 [42], observational (n = 742)	Dhaka, Bangladesh, urban tertiary hospital, PICU. Nurse: patient ratio not reported, review frequency not reported, doctor presence not reported, CPAP training details not reported, CXR availability not reported, NGT use not reported	<5 y, WHO severe pneumonia and SpO2 < 90% in room air. Excluded: presented with features of respiratory failure (eg, gasping) or congenital heart disease. Median age 7.3 mo, mortality 5.7%	Bubble CPAP	None	Mortality	Mortality: 5.7% CPAP vs 21% in children with same eligibility criteria.	Not reported
Jayashree 2016 [43], observational (n = 330)	Chandigarh, India, urban tertiary hospital, emergency unit. Nurse: patient ratio not reported, review frequency: 2 hourly, doctor presence not reported, CPAP training details not reported, CXR available, NGT use not reported	1m-12years, clinical pneumonia and hypoxaemia Excluded: GCS<10, asthma, upper airway obstruction, congenital heart disease, pneumothorax. Pneumonia 73%, bronchiolitis 27%, Median age 8.4 mo, mortality 2.7%	Bubble CPAP	None	Need for intubation	163 patients given CPAP, of which 3 (1.8%) required intubation, 1 (0.6%) of whom died.	Total: 16 (9.8%), 7 (3.8%) hypercarbia, 3 (1.8%) pneumothorax, 3 (1.8%) nasal trauma, 3 (1.8%) abdominal distension
McCollum 2019 [36], RCT (n = 664)	Salima, Malawi, rural district hospital. Nurse: patient ratio not reported, review frequency: 6 hourly vitals and nasal interface review, twice daily full clinical evaluation. Review 1 h after CPAP change. Doctor presence: no doctors present on site. Telephone consultation available. CPAP training: 5-d training period, twice-annual refresher courses; on-site paediatrician supervision every 2 weeks. Tested on clinical evaluation of patients, use of decision algorithms, and decision making during directly observed care. Remediation. All study nurses had at >1 y of bCPAP experience at the referral hospital, the nurse coordinator had 5 y experience. Portable CXR not available. CXR if persistent or worsening illness (if safe). Per protocol exclusive NG feeding if general danger sign, apnoea, grunting, >2 respiratory danger signs. In practice: 96% of children enrolled met criteria, only 16% received NG feeds (CPAP 7% less frequent vs low flow).	1-59m, WHO severe pneumonia and one or more high-risk conditions (HIV infection or exposure, severe malnutrition, SpO_2_<90%). Excluded: Re-presented after having already been enrolled and discharged. Median age 7.6 mo in CPAP arm, 7.7 mo in low flow O_2_ arm, mortality 14%.	Bubble CPAP	Low flow oxygen	Survival to discharge	Mortality: 17% (n = 53) CPAP vs 11% (n = 35) low flow, RR = 1.52, *P* = 0.036. Excess mortality of CPAP detected in some subgroups (SpO_2_<90% RR = 1 · 65, *P* = · 048, age <12 mo RR = 1.70, *P* = 0.049, Blantyre Coma Score ≥5 RR = 1.63, *P* = 0.049, not severely anaemic RR = 1.53, *P* = 0.048, no diarrhoea RR = 1.53, *P* = 0.040). However, no significant interaction with intervention for any clinical variable detected.	Total: 3% (n = 11) CPAP vs <1% (n = 1) low flow oxygen. 4 CPAP and 1 oxygen group death were classified as probable aspiration episodes. 1 CPAP death classified as probable pneumothorax. 6 non-death CPAP events included skin breakdown around the nares.
**Respiratory failure**							
Kinikar 2011 [44], observational(n = 36)	Pune, India, urban tertiary hospital, ICU. Nurse: patient ratio not reported, review frequency: 0 and 6 h, doctor presence not reported, CPAP training details not reported, CXR routine 1-4 h after starting CPAP, respiratory distress scoring, NGT use not reported	<12 y, influenza-like illness with respiratory distress (dyspnoea, tachypnea, accessory muscles) with FiO_2_>40% to maintain SpO_2_>94%. Excluded: Shock refractory to volume/ dopamine, apnoea, Pao_2_/FiO_2_<200. Pneumonia 90%, empyema 10%, median age 18 mo. No patients died	Bubble CPAP	None	Improvement in vital signs and gas exchange over first six hours	Respiratory rate and heart rate fell in the first six hours of CPAP. Pao_2_/FiO_2_ improved in 6 h (*P* < 0.017). pH pre 7.3 Post 7.4, *P* < 0.0001. Pco_2_ pre 43, post 33, *P* < 0.0001. Po_2_ pre 65, post 239, *P* < 0.0001 SpO_2_ pre 87 (82-89), post 97 (96-99), *P* < 0.001. No patients required intubation	None reported (including nasal and facial skin erosions, conjunctivitis, gastric distension, and air leakage).
Myers 2019 [46], observational (n = 117)	Lilongwe, Malawi, urban tertiary hospital, emergency and HDU. Nurse: patient ratio 1:15-1:30, review frequency: vital signs 2-3 times per day, spot checks 2-4 hourly in HDU, doctor presence: 2-4 doctors or clinical officers present during day, 1-2 at night, at least one consultant (senior doctor) on site for 12 h per day. CPAP training details not reported. Basic bedside ultrasound, echocardiography available. CXR availability inconsistent. NGT insertion for initial gastric decompression and drainage	<15 y, critically ill, severe respiratory distress after initial resuscitation. Pneumonia 53%, multiorgan failure 47%, median age 7 mo, mortality 32.5%	Bubble CPAP	None	Survival to discharge	Survival to discharge: 67.5% (n = 79), 45% (n = 34) with multiorgan failure, 36% (n = 10) with severe acute malnutrition	11% (n = 13) of patients had equipment-related complications. 2 blocked nostrils 2 blocked nasal prongs. 2 interruption of oxygen supply from depleted cylinder. 7 nasal septum lesions. 1 aspiration of feeds
Pulsan 2019 [47], observational (n = 64)	Port Moresby, Papua New Guinea, urban tertiary hospital. Nurse: patient ratio usually 1:4 but variable, review frequency: Vital signs at 0, 1 h, then 12 hourly. Continuous pulse oximetry monitoring for patients in ICU. Doctor presence: paediatric registrar present during day and on-call 24 h (covering ICU and three wards). Paediatrician ward round each morning, and 4-5 pm; on-call otherwise. CPAP training: nurses and doctors trained in CPAP including indications, administering required flow rate and oxygen, monitoring of patients, and care of CPAP machines. CXR available (72% had CXR by enrolment). Enteral nutrition via a NGT when safe to do	Severe acute lower respiratory infection, with SpO_2_<90% or severe respiratory distress despite standard oxygen therapy Excluded: congenital heart disease, chronic lung disease, severe anaemia in heart failure, newborns with severe birth asphyxia. Pneumonia 75%, TB 7.8%, neonatal RDS 3%, bronchiolitis 1.6%, median age 3 mo, mortality 54.7%	Bubble CPAP	None	Change in O_2_ saturations and respiratory distress scores, and mortality	Median SpO_2_ increased from 78% to 92% after one hour of CPAP. Median respiratory distress score improved in one hour from 11 to 9. Number of patients with SpO_2_<85% decreased from 61% (n = 39), to 29.7% (n = 19) after one hour. All those who survived maintained SpO_2_>85. SpO_2_ 1 h after CPAP commencement was strongest predictor of survival	Not reported
Walk 2016 [50], observational (n = 77)	Lilongwe, Malawi, urban tertiary hospital. Nurse: patient ratio 1:6 in HDU, 1-2:40-70 in emergency department, review frequency: at least twice daily, doctor presence: a clinical officer, medical intern or qualified doctor staffed the ward. CPAP training details not reported. CXR availability not reported. NGT for feeding and stomach decompression in all patients.	All patients aged 1 week to 14 y with acute respiratory failure who were deteriorating on standard treatment with antibiotics and oxygen. Pneumonia 86%, respiratory depression 10%, median age 5 mo, mortality 47%	Bubble CPAP	None	Death or endotracheal intubation with manual ventilation (mechanical ventilation not available)	47% mortality. Age 0-2 mo 93% lower odds of CPAP failure than age >60 mo of age (OR = 0 · 07), other age groups not significantly different. Children with HIV (OR = 4.35), co-morbidity (OR = 3 · 52) or multi-organ failure (OR = 12 · 67) had higher odds of CPAP failure than those who did not	Total: 16.9% (n = 13), 11.7% (n = 9) nosebleeds, 3.9% (n = 3) other nasal, 2.6% (n = 2) airway obstruction requiring suctioning. Drying of nasal mucosa in all patients requiring saline drops
**Case studies**							
Brown 2013 [53], case reports (n = 2)	Blantyre, Malawi, urban tertiary hospital, paediatric ward. Nurse: patient ratio not reported, review frequency not reported, doctor presence not reported, CPAP training details not reported, CXR availability not reported, NGT use not reported	N/A – one neonate and one 6m old.	Bubble CPAP	None	N/A – case report	2 patients successfully treated	No evidence of mucosal drying or other complications were observed
Larsen 2020 [54], case report (n = 1)	Korogwe, Tanzania, rural district hospital. Review frequency not reported, NGT not used, patient monitored for gastric distension	N/A – case report of 6m old	Bubble CPAP	None	N/A – case report	Some improvement in vital signs following initiation of treatment. Discharged in good condition after 5 d	Air leakage causing failure to reach desired pressures, resolved by improving seal through use of tape around nasal cannulae.
McCollum 2011 [55], case report (n = 1)	Lilongwe, Malawi, urban tertiary hospital, HDU. Review frequency not reported, under paediatrician supervision. all nurses trained in use of CPAP. CXR performed. Orogastric tube placed	N/A – case report of 3m old infant	Bubble CPAP	None	N/A – case report	Initial increase in oxygen saturation. Weaned to room air after 7 d. Subsequent discharge in good condition	Not reported
**Other non-clinical implementation outcomes**							
Kortz 2017 [52], cost-effectiveness analysis	Malawi, national level analysis N/A	N/A	Bubble CPAP	None	Predicted cost compared to standard care, and incremental cost-effectiveness ratio per disability-adjusted life year (DALY) averted	Cost of treating one child with severe pneumonia US$88 for standard of care and US$152 with bCPAP (incremental net cost of US$64 per use of bCPAP).CPAP averts 5.0 DALYs per child treated, incremental cost-effectiveness ratio of US$12.88 per DALY averted.	N/A
Sessions 2019 [48], observational (n = 40)	Salima, Malawi, rural district hospital. N/A – see McCollum 2019	1-59m, WHO severe pneumonia and one or more high-risk conditions (HIV infection or exposure, severe malnutrition, SpO_2_<90%). Mean age 11.35 mo	Bubble CPAP	None	Time spent administering CPAP or oxygen and associated costs	In first 4 h, average of 34.71 min longer, per patient, initiating CPAP compared to low-flow oxygen. Nasal CPAP interface adjusted an average of 9.17 times vs 2.55 time low flow. No significant difference in 48 h of maintenance of therapy, though more time spent adjusting equipment 4.57 vs 1.52, *P* = 0.04.	N/A – see McCollum 2019
Sessions 2020 [49], observational, qualitative (n = 54)	Salima, Malawi, rural district hospital. N/A – see McCollum 2019	All mothers whose child had been enrolled in CPAP IMPACT (McCollum 2019)	Bubble CPAP	None	Maternal beliefs about CPAP and oxygen	Widespread negative beliefs about oxygen and CPAP in the community, anxiety and fear surrounding care before initiation of treatment, and a shift in beliefs to a more positive opinion of treatment regardless of clinical outcome	N/A – see McCollum 2019
Wilson 2014 [51], observational	Kintampo, Mampong, Nkoranza, and Wenchi, Ghana, 4 rural hospitals. N/A – see Wilson 2013	Hospitals which participated in Wilson 2013	Bubble CPAP	None	State of staff knowledge and skills and state of CPAP equipment 16 mo following initial study	Seven of eight CPAP machines were present and functional. 5/8 oxygen concentrators and 3/4 generators were non-functional. First-generation trainees scored significantly higher than second-generation trainees on both skills and knowledge assessments	N/A – see Wilson 2013

ARDS – acute respiratory distress syndrome, CPAP – continuous positive airway pressure, CXR – chest radiography, GCS – Glasgow Coma Score, HDU – high dependency unit, HFLC – high-flow nasal cannula, LFNC – low-flow nasal cannula, NGT – nasogastric tube, PICU – paediatric intensive care unit, PJP – pneumocytis jiroveci pneumonia, PSNZ – Paediatric Society of New Zealand, RCT – randomised controlled trial, RDS – respiratory distress syndrome, TB – tuberculosis, RR – relative risk, OR – odds ratio, CI – confidence interval

### Risk of bias

Of the 19 studies providing original clinical data, quality was assessed as weak in 7 studies [33,40,41,44,53-55], moderate in 10 [34,35,37,38,42,43,45-47,50] and strong in 2 studies (Table S2 in the **Online Supplementary Document**) [36,39].

### Where were the studies conducted?

Studies took place in 2 low-income countries (Uganda and Malawi), 5 lower-middle income countries (Bangladesh, India, Ghana, Papua New Guinea, and Tanzania) and 1 upper-middle income country (Brazil) (**Table 1**).

Fourteen (61%) studies were predominately in urban tertiary hospitals [33-35,37,40,42-47,50,53,55], and 8 (35%) were in district level hospitals (mostly rural) [36,38,39,41,48,49,51,54]. Of the 14 tertiary hospital based studies, 6 took place in intensive care wards [33,34,37,40,42,44].

A total of 5050 children were enrolled in the 19 included studies that provided original clinical data (including case reports): 43.5% (2200) in a single large crossover study in two district hospitals in Ghana [39]. This study aside, 72% (2052/2850) of the remaining children were studied in tertiary centres.

### Which children have been studied?

While all included studies included a subset of children with pneumonia requiring CPAP, the populations included children of varying ages with bronchiolitis, all cause respiratory distress, respiratory failure, as well as WHO-defined severe or hypoxaemic pneumonia (**Table 1**).

Three randomised trials focussed on infants aged <12 months with bronchiolitis (total 165 participants), all conducted in tertiary hospitals settings in Brazil and India, including two in paediatric intensive care units (PICU) [33,35,37]. These studies all reported zero inpatient mortality in both CPAP and comparison groups (high-flow or standard oxygen therapy), small or no improvement in clinical signs, and similar rates of treatment failure and length of stay between CPAP and High Flow Nasal Cannula (HFNC) or standard care groups. Young infants with likely bronchiolitis were also reported in observational studies of WHO pneumonia in India (27%-31% of total population) [40,43], Malawi (53% of total population) [45], Papua New Guinea (1.6% of total population) [47], and in 3 case reports of successful CPAP treatment in Malawi and Tanzania [53-55].

Five studies included children with general respiratory distress (total 2763 participants), including 3 observational studies [40,41,45] and 2 trials comparing CPAP to delayed initiation of CPAP or standard care [38,39]. These studies included a range of ages and illnesses, with mortality ranging from 3% (typically younger infants with bronchiolitis) to 29% (high HIV-related pneumocystis carinii pneumonia). Three comparative studies from semi-rural hospitals in Ghana and Uganda suggested that CPAP was associated with respiratory improvement within a few hours of initiation [38,41], and possible mortality benefit for infants <1 year of age but not older children [39].

Two trials [34,36] and two observational studies [42,43] focussed on children with hypoxaemic or high-risk pneumonia (total 1967 participants), with mortality ranging from 3% to 14%. The trial from a tertiary PICU in Bangladesh reported reduced mortality with CPAP compared to high-flow or standard low-flow oxygen [34], but the trial in a rural district hospital in Malawi found increased mortality [36]; both trials were stopped early at interim analysis.

Four observational studies enrolled children with respiratory failure (failing standard oxygen therapy), mostly related to severe pneumonia. Mortality ranged from 33% to 55% (urban Papua New Guinea and Malawi) [46,47,50], except for one Indian study involving children with influenza-like illness during the swine flu epidemic (zero mortality) [44]. These studies reported improved respiratory signs and did not include a comparison group.

Across all these studies, older children (especially >12 months), children with HIV, severe acute malnutrition, multi-organ failure, or other comorbidities generally fared worse on CPAP that those without [39,46,50]. Early response to CPAP (specifically correction of hypoxaemia) was a strong predictor of survival [47].

### CPAP set-up

All but 3 of the studies [33,37,40] used a form of bubble CPAP (**Table 2**). Seven studies used commercially available bubble CPAP systems [36,38,39,45-47,53]. In 5 studies a bubble CPAP circuit was improvised by using nasal prong tubing, cut with one limb submerged under a column of water, the depth determining set pressure [41,56-59]. Depths between 5 and 10 cm were used in included studies. Other modalities included a flow-inflating device [40], and CPAP via a mechanical ventilator [33,37].

**Table 2:** CPAP details of included studies

Paper	CPAP Type	O_2_/Air source	Blender mechanism	Circuit tubing and nasal interface	Bottle used for insertion of expiratory limb	Humidification method	Commercially available or improvised	Equipment cost (USD)*
Anitha 2016 [40]	Flow inflating device Mapleson D (Bain circuit) or Mapleson F (JR circuit)	Not specified	None	Mask	N/A	None	Commercially available	-
Bjorkland 2018 [41]	Bubble. Adapted LFNP oxygen tubing, cut with one limb submerged under column water	Cylinder or oxygen concentrator	None	Standard paediatric nasal cannulae and tubing, with cut 'ear plugs' around nasal prongs to provide seal	Disposable water bottle	None	Improvised	US$5
Brown 2013 [53]	Bubble. Integrated device: Flow driven, 2 air pumps plus O_2_ source.	Cylinder or oxygen concentrator	Integrated	Nasal prongs	Integrated	None	Commercially available	US$350
Cesar 2018 [33]	Drager Evita 4 ventilator	Central	Integrated	Fitted nasal mask	-	Integrated	Commercially available	-
Chisti 2015 [34]	Bubble	Oxygen concentrator (Airsep)	Oxygen concentrator (Airsep)	Standard nasal oxygen prongs (Ventlab) and intravenous fluid tubing (Opso Saline)	Transparent shampoo bottle	-	Improvised	-
Chisti 2018* [42]	Bubble	Oxygen concentrator (Airsep)	Oxygen concentrator (Airsep)	Standard nasal oxygen prongs (Ventlab) and intravenous fluid tubing (Opso Saline)	Transparent shampoo bottle	-	Improvised	-
Jayashree 2016 [43]	Bubble	Central	None	Nasal prongs	Glass bottle	None	Improvised	-
Kinikar 2011 [44]	Bubble. Adapted LFNP oxygen tubing, cut with one limb submerged under column water	Oxygen concentrator	Oxygen concentrator	Paediatric nasal cannula	Normal saline bottle	None	Improvised	250 Rs = US$3.40†
Lal 2018 [35]	Bubble	Cylinder	Y-tube connecting O_2_ and Air sources	Nasal prongs or nasopharyngeal catheter		Heating element with water that keeps air passing over it saturated with moisture at body temperature	Improvised	With humidifier: 5000 Rs = US$68†. Without humidifier: 500 Rs = US$6.80†
Larsen 2020 [54]	Bubble. Adapted LFNP oxygen tubing, cut with one limb submerged under column water	Oxygen concentrator	Oxygen concentrator	Infant nasal cannula and prongs, switched to child size on day 2, sealed with tape wrapped around each cannula on day 3	Sterile water bottle	Oxygen concentrator	Improvised	-
Machen 2015 [45]	Bubble. Integrated device: Flow driven, 2 air pumps plus O_2_ source.	Cylinder or oxygen concentrator	Integrated	Nasal prongs	Integrated	None	Commercially available	US$350
McCollum 2011 [55]	Bubble. Adapted LFNP oxygen tubing, cut with one limb submerged under column water	Oxygen concentrator (Airsep)	Oxygen concentrator (Airsep)	Size 4 Hudson nasal prongs	Container of water	Oxygen concentrator	Improvised	
McCollum 2019 [36]	Bubble. Validated bCPAP system (Fisher and Paykel)	Oxygen concentrator (Airsep)	Oxygen concentrator (Airsep)	Either unvented nasal masks or nasal prongs	Integrated system's water reservoir	Integrated	Commercially available	
Myers 2019 [46]	Bubble. Modified oxygen concentrators (Diamedica)	Oxygen concentrators	Oxygen concentrators	Nasal prongs	Integrated	Integrated	Commercially available	
Pulsan 2019 [47]	Bubble. Modified oxygen concentrators (Diamedica)	Oxygen concentrators	Oxygen concentrators	Low resistance tubing and low-resistance nasal oxygen prongs	Integrated	Integrated	Commercially available	
Sarkar 2018 [37]	CPAP via a mechanical ventilator (SERVO-i®)	Central	Integrated	Nasal prong or nasal mask (SERVO-i®)	N/A	Integrated	Commercially available	
Walk 2016 [50]	Bubble (nasal prong tubing, cut with one limb submerged under column water)	Oxygen concentrator	None	Binasal prongs or nasopharyngeal tube	Water bottle	None	Improvised	
Wilson 2013 [38]	Bubble CPAP (DeVilbiss IntelliPAP CPAP machine)	Central or concentrators	Integrated	Hudson RCI CPAP nasal cannula	Closed system. 0.0025% acetate solution in water	Integrated	Commercially available	Approx US$400 for CPAP set up, US$32 for disposable nasal prongs
Wilson 2017 [39]	Bubble CPAP (DeVilbiss IntelliPAP CPAP machine)	Central or concentrators	Integrated	Three sizes of single-use Hudson RCI nasal prongs	Closed system. 0.0025% acetate solution in water	Integrated	Commercially available	Assume same as Wilson 2013 above

CPAP – continuous positive airway pressure

*Cost of CPAP equipment only excluding oxygen provision and ongoing maintenance costs.

†Exchange rate Indian rupee: US$, 74:1.

Oxygen was supplied by oxygen concentrators or cylinders in most studies, with wall or central pressurised O_2_ available in 3 studies [38,39,43]. Fifteen systems allowed blending of air and oxygen, 10 included humidification, and only one heating [35]. Two studies used mask interfaces [33,40]. The remaining used exclusively nasal prongs, a combination of prongs and nasopharyngeal catheters [35,50] or prongs and masks [36,37].

### Staffing and clinical practice context

Of 19 studies providing original clinical data, only 3 observational studies from urban hospitals in Africa reported nurse to patient ratios (range = 1:2 to 1:40) (**Table 1**) [46,47,50]. Similarly, few studies reported doctor supervision, generally involving a mid-level doctor or medical officer available onsite during the day and on-call overnight with at least daily senior medical ward round [39,46,47,50]. The notable exception was one trial in rural Malawi that was nurse-led (doctor available by phone for advice only) and was stopped early due to excess mortality in the CPAP arm [36].

Studies typically required nursing review within 1-2 hours of commencing CPAP or changing settings [34-41,43,45,47], then just 2-4 times per day [36,38,39,41,44-47,50], with only three studies from tertiary hospitals in India and Bangladesh continuing 1-2 hourly observations [34,40,43]. Many studies used pulse oximetry [33-44,46,47,50], and sometimes blood gas analysis and/or formal severity scores [34,35,37,41,43,44,47], to monitor clinical response, but frequency of monitoring was rarely documented.

Nasogastric or orogastric tubes (NGT/OGT) for gastric decompression and/or feeding were commonly included in study protocols but adherence to this was poorly reported [34,36,37,40,41,46,47,50,54,55]. In one study although 96% of patients met criteria for NG tube insertion, only 16% received one [36]. Chest radiography (CXR) was available in most facilities [34-37,41,43,44,47], but not all [39,46].

### Equipment and training

Training was typically directed at nurses [34,36-39,41,47], often described as interactive/hand-on/practical [36,38,41], included some test of competence [36,38,41], and follow-up supervision or re-training [36,38,39].

Few studies reported on equipment function, maintenance, or sustainability, and those that did found practical challenges. An RCT from a PICU in Bangladesh reported equipment failure in 20% of cases during the trial [34]. During a crossover trial in 2 small hospitals in Ghana, CPAP was stopped early due to loss of power in 1 patient despite backup generators provided by the study team [39]. Six patients ran out of oxygen while on CPAP during an observational study in a regional hospital in Uganda [41].

Even less is known about medium- and long-term sustainability of equipment or health care worker skills. A 16-month follow-up study of the Ghana trial found that 13% (1/8) of CPAP machines and 63% (5/8) of oxygen concentrators were non-functional and detected a significant drop-off in skills and knowledge in the staff who had been trained since the end of the study (despite some retraining having taken place) [51]. A case report from rural Tanzania highlighted challenges to sustained CPAP practice due to unavailability of components and increased demands on clinical staff [54].

### Comfort and complications

CPAP adverse effects were inconsistently reported, with minor complications (eg, mucosal dryness, nasal skin trauma, mild gastric distension) ranging from <10% [35,36,38-41,43,44,46] to >50% [37,50] of participants. Studies that compared CPAP to HFNC or low-flow oxygen generally found higher rates of minor complications with CPAP [36,37]. Aspiration (0%-1.2%) [34,36,41,46] and pneumothorax (0%-1.8%) [34-39,41,43,44] events were uncommon.

### Cost

Costs were reported in 7/19 studies providing clinical data: three studies reported the costs of modifying an existing oxygen (+/− air) source to provide bubble CPAP (range US$3.40 to 6.80 without humidifier) [35,41,44], and four studies reported the cost of a low cost CPAP device and consumables (US$350 to 400) – but none included personnel, training, or maintenance costs (**Table 2**) [38,39,45,53]. In a cost-effectiveness analysis, Kortz inputted one-off and ongoing CPAP equipment and costs, training costs, but not costs for extra personnel or staffing time [52]. In this analysis CPAP for severe pneumonia cases costed US$64 per patient more than standard care, with a favourably cost per Disability Adjusted Life Years (DALY) averted compared to other pneumonia interventions.

Reporting of the personnel and time-cost of CPAP is a gap in the literature. Recent work from Sessions et al found that health care workers performed more tasks and took more time (118 vs 83 minutes to initiate and 4.6 vs 1.5 minutes to adjust) with CPAP treatment relative to low flow oxygen [48]. They estimated this could lead to an additional 164 hours of work per month hospital if CPAP use was expanded to all patients with severe pneumonia (with or without comorbidity).

## DISCUSSION

This review aimed to understand patient selection and contextual factors that influence the effectiveness and safety of CPAP for children with severe respiratory infection that would meet WHO’s definition of severe pneumonia. We found heterogeneity in patient population, contexts, and intervention that limits the ability to pool results. However, while there are gaps, there are currently sufficient data and expert opinion to outline practical recommendations.

### Which children benefit from CPAP?

Our understanding of the mortality benefits of CPAP for severe respiratory distress in children in low and middle income countries has been shaped by 3 RCTs demonstrating significant benefit in reducing clinical failure and mortality [34], no benefit in all-cause mortality [39], and increase in mortality [36]. Hypotheses behind the reasons for these conflicting results highlight the importance of context and study design in assessing the potential role of CPAP [60-62].

In this review we document the heterogeneity of patient populations studied across both RCTs and observational studies, reflected in the large differences in the proportion of children who died in the studies. The reasons are multifactorial, but importantly reflect differences in case definition, inclusion and exclusion criteria. Predictably, studies of bronchiolitis (ie, single organ pathology) based in intensive care or highly monitored units, with access to mechanical ventilation, had the lowest (zero) mortality. Conversely, studies that enrolled children with respiratory failure – defined as hypoxaemia or respiratory distress worsening or not improving with low flow oxygen – documented mortality in 50% of children. So, while all studies enrolled patients that would fulfil WHO’s definition of severe pneumonia this encompasses a wide range of populations from infants with (mostly) self-limiting bronchiolitis to older children with complicated pneumonia and multi-organ failure [63].

This review highlights that our evidence base for post-neonatal CPAP is heavily skewed by data in young children, with most studies enrolling children with mean/median ages <18 months. This is reflected in both studies exclusively of bronchiolitis [33,35,37], and in studies of pneumonia enrolling a significant proportion (27%-54%) of infants with bronchiolitis [40,43,45]. In others, it reflected the higher risk of severe disease and deterioration in the first year of life [47,50].

**Table 3** summarises the current evidence using the Oxford Levels of Evidence framework [10]. In keeping with studies from high resource settings [35], there is evidence that CPAP reduces respiratory distress and improves oxygenation in bronchiolitis, and possibly reduces mortality. The current literature most supports the use of CPAP in infants <1 year with likely bronchiolitis, who have hypoxaemia or respiratory distress not responding to low flow oxygen and preserved respiratory drive and upper airway reflexes, in settings with low HIV prevalence and malnutrition and adequate staffing. For this population, it would make sense to align (at least the CPAP set-up) with what is being recommended for neonatal CPAP [64,65]. **Box 1** provides an example checklist to assess appropriateness of implementing CPAP in any given setting.

**Table 3:** Conclusions from included studies

Conclusions	Key supporting results	Level of evidence
CPAP reduces signs of respiratory distress in children (<5 y) with respiratory distress due to lower respiratory tract infections.	Improved SpO_2_ [41,44,45,47,50]. Reduced respiratory rate [38,40,41,44,45,50]. Improved respiratory distress score [41,45].	3
CPAP reduces signs of respiratory distress in infants with bronchiolitis.	Improved SpO_2_ [45]. Reduced respiratory rate [35,40,45]. Improved respiratory distress score [35,45].	2
CPAP reduces mortality in infants (<1 y) with respiratory distress, preserved respiratory drive and upper airway reflexes.	All-cause mortality 10/374 (3%) CPAP vs 24/359 (7%) control RR = 0.4 (0.19-0.82, *P* = 0.01) [39].	2
CPAP reduces treatment failure in children <5 y with WHO-defined severe pneumonia and hypoxaemia in an ICU setting.	Treatment failure: 5/79 (6%) CPAP vs 16/67 (24%) low flow RR = 0.27 (0.07-0.99, *P* = 0.0026) [34].	2
CPAP reduces mortality in children <5 y with WHO-defined severe pneumonia and hypoxaemia in an ICU setting.	Death: 3/79 (4%) CPAP vs 10/67 (15%) low flow RR = 0.25 (99.7% CI = 0.07-0.89, *P* = 0.02) [34].	3
CPAP may have no impact or increase mortality of children <5 y in settings with limited medical supervision and low nurse to patient ratios.	Death: 26/1021 (3%) CPAP vs 44/1160 (4%) low flow RR = 0.67 (0.42-1.08, *P* = 0.11) [39]. Death: 53/321 (17%) CPAP vs 35/323 (11%) low flow RR = 1.52 (1.02-2.27, *P* = 0.036) [36]. Meta-analysis (death): RR = 0.75 (95% CI = 0.33-1.72) [8] (includes [36,39] and Chisti [34]).	2
Bubble CPAP is safe, with low risk of serious adverse events with regular nursing oversight.	Low incidence of adverse events [34-36,39,41,43].	2
There is insufficient evidence to determine whether HFNC is non-inferior to CPAP for infants with bronchiolitis in low resource settings.	Treatment failure: 10 (35.7%) CPAP vs 13 (38.2%) HFNC, *P* = 0.952 [33]. Treatment failure: 6% CPAP vs 13% HFNC, RR = 0.50 (99.7% CI = 0.11-2.29), *P* = 0.175 [34]. Mortality: 4% CPAP vs 13% HFNC, RR = 0.30 (99.7% CI = 0.09-1.05), *P* = 0.082 [34]. No differences in length of hospital stay, length of stay, or duration of respiratory support needed [33,35].	2
Units offering CPAP need to have daily medical oversight.	Effectiveness in ICU setting [34]. Harm in settings with no medical supervision [36]. Safety in district hospital setting with daily medical oversight [38,39].	2
CPAP should not be provided as an additional therapy without increasing nurse to patient ratios in non-ICU/HDU settings.	Significant nursing time for CPAP set-up and observations [48]. Harm in under supervised / high patient to nurse ratio setting [36,46].	3
Children on CPAP should have a nasogastric inserted, and allowed to vent to prevent stomach over-distension.	Potential aspiration events [36]	3
Children on CPAP need to have a feeding plan – nil by mouth until determined safe to feed by supervising physician.	Potential aspiration events [36]. Safety when clear protocols in place [39,47,60].	3

Box 1 **Assessing suitability of CPAP**

Below are some questions to assess whether CPAP would be a suitable intervention in each setting. If the answer to any of these questions is no, it should be addressed prior to reconsidering.

1. Are there adequate numbers of staff day and night so that patients on CPAP:

- are continuously monitored with adjustments made to the CPAP as needed to maintain efficacy (eg, maintaining an adequate nasal seal to maintain pressures);

- will receive any additional necessary management required (eg, nasogastric tube for decompression of stomach distension and nasogastric feeding or intravenous fluids);

- clinical deterioration will be quickly identified, and appropriate steps taken

2. Are all nursing and medical staff who will be caring for patients on CPAP:

- adequately trained in CPAP use, including knowledge of indications for use, how to set up CPAP, how to maintain it

- aware of the potential complications (eg, pneumothorax, nasal pressure areas, risk of unrecognised respiratory failure) and have skills in how to recognise and manage such complications safely and effectively

- is there a plan for ongoing monitoring and training to account for loss of knowledge and skills and / or staff turnover

3. Is adequate equipment available for using CPAP?

- Ensure CPAP will not negatively impact on care of other patients (eg, are there sufficient oxygen sources available that giving one patient CPAP will not limit access to other patients to standard oxygen therapy)

- Ensure infection control and equipment sterilisation procedures are in place

- Ensure technician/health care worker trained in day-to-day trouble shooting and maintenance

- Ensure adequacy of power supply or back-up source of power

4. Are adequate monitoring tools available?

- Monitoring charts of vital signs with thresholds for responses and escalation

- Clinical guidelines on the use of CPAP and care of patients with acute respiratory disease

- Pulse oximetry

- Audit processes

### Research directions

Future research should aim to further understand the subset of patients, fulfilling WHO’s definition of severe pneumonia and presenting outside of tertiary and intensive care units, who would most benefit from CPAP. And to determine clinical predictors that can be used to identify these children to further clarify guidelines on eligibility for safe and effective CPAP. Conversely, it would be also important to predict subsets of children who would do badly on CPAP, and understand the aetiology of CPAP failure or death. Building on the evidence base of minimal standards to sustain safe and effective CPAP will assist wider adoption. Research on CPAP use should ideally report all above reviewed contextual factors to provide readers with a better understanding of the intervention, the environment in which it was introduced, and the population in whom it was used. Ideally, costs should include total systems cost (including staffing, training, consumables, power, maintenance and repairs), and not just equipment costs, so that facilities can evaluate feasibility and cost-effectiveness in considering CPAP adoption. When CPAP is introduced into a setting significantly different from those studied in the literature, it is best to do so in the context of a quality improvement evaluation, such that efficacy can be properly reviewed in that setting prior to wider uptake. An evaluation and reporting framework for CPAP studies has been proposed (**Table 4**) [66].

**Table 4:** Reporting framework of CPAP studies adapted from Duke 2019 [61]

CPAP studies should include objective reporting of the characteristics of their equipment, method, patient group, staffing, monitoring and supportive care, equipment maintenance requirements and outcomes.	
CPAP equipment	• Type of CPAP – ventilator, commercial or improvised bubble CPAP; gas flow source for oxygen and air; blender and humidifier method; circuit tubing diameter and composition. If improvised, details of container used for insertion of expiratory limb; interface – mask or prongs, brand and size.
CPAP clinical method	• Pressure – starting, maximum and titration guidelines; monitoring of whether pressure is delivered (bubbling); flow – gas/oxygen flow rates used; fraction of inspired oxygen – starting and maximum.
Patient group / inclusion criteria	• Age; disease/condition – including differentiation of pneumonia and bronchiolitis; eligibility for CPAP – eg, respiratory distress score, or hypoxaemia despite low flow oxygen; comorbidities;exclusion criteria – eg, GCS<8, persistent vomiting.
Monitoring and supportive care	• Pulse oximetry – frequency, and response to hypoxaemia; monitoring/observation charts – frequency of observations and defined responses and escalation procedures; respiratory distress score; fluids and feeding management; nasogastric insertion, free drainage; guidelines for management of childhood illness and CPAP.
Health service aspects	• Setting – urban/rural, tertiary/district, government/private, general ward/high dependency unit/separate intensive care unit; nurse – patient ratio, day, and night; paediatrician presence; doctor presence 24 h a day; nurse training in acute care and CPAP; details on CPAP training for nursing and medical staff; chest x-ray facilities; frequency of ward rounds.
CPAP maintenance	• Biomedical technician trained to repair CPAP machines and other oxygen equipment; oxygen analyser for checking performance of oxygen equipment.
Outcomes	• Mortality rates – overall and disease/age specific (causes of death); adverse events (pneumothorax, nasal bleeding or pressure areas, aspiration).
Cost	• Equipment; additional personnel; running costs – including maintenance/repairs, power, consumables.

### Limitations

Our review was intentionally broad, to capture all CPAP-related studies from low- and middle-income countries, and enabled us to capture 7 studies not included in previous systematic reviews [8,9]. We restricted our search to CPAP, rather than other non-invasive ventilation (NIV) strategies, to make our findings most applicable to low-resource settings where low-cost CPAP is likely to be more feasible than strategies requiring a mechanical ventilator. We also limited our search to English-language articles and we are aware that this excluded some studies from Latin America [67,68]. Despite this, our review included a broad range of settings across Africa and Asia-Pacific regions and the findings will be highly relevant to health managers and policy-makers who are seeking to enhance hospital care for children with severe pneumonia.

## CONCLUSIONS

CPAP is a safe intervention in settings resourced with intensive monitoring and supportive care, and the strongest evidence for a benefit of CPAP is in infants (less than 1 year) with bronchiolitis. The available published evidence and clinical experience can be used to help facilities assess appropriateness of implementing CPAP, guide health workers in refining selection of patients most likely to benefit from it, and provide a framework for components of safe and effective CPAP therapy. There is scope for future research to better detail and standardise the reporting of context, studied populations and CPAP set-up to assist policy makers and health workers in adapting research findings to their local setting.

## Additional material


Online Supplementary Document.
