# Supplementary material for: Continuous Positive Airway Pressure (CPAP) for severe pneumonia in low- and middle-income countries: A systematic review of contextual factors
Source: J Glob Health. 2022 Oct 30;12:10012. doi: 10.7189/jogh.12.10012 (PMC9586144; doi:10.7189/jogh.12.10012)
Supplement: Online Supplementary Document. [file jogh-12-10012-s001.pdf]

ONLINE SUPPLEMENTARY DOCUMENT

Title: Continuous Positive Airway Pressure (CPAP) for severe pneumonia in low- and middle-income countries: a systematic review of contextual factors

Authors: Chris Wilkes, Rami Subhi, Hamish Graham, Trevor Duke, on behalf of the ARI Review group

Contents

Appendix S1 – Further details of study methods including search strategy, data extraction and data management 2

Appendix S2 – MEDLINE database search 6

Table S1. Inclusion and exclusion criteria for studies in this review 8

Table S2. EPHPP quality assessment of included studies 9

Appendix S1 – Further details of study methods including search strategy, data extraction and data management

PICOTS

- Population - Children aged 28 days-5 years with pneumonia defined according to WHO criteria.
- Intervention - CPAP (any form)
- Comparison - usual care, low-flow, or high flow oxygen
- Outcomes addressed - acceptability, feasibility, safety (unintended effects, practice points, required capacity) and / or efficacy (change in clinical signs, rates of treatment failure, length of stay, and mortality)
- Time period – 2000 to present
- Setting - hospitals (secondary and tertiary) in low and middle income countries (as defined by the World Bank)
- Study types – All relevant published randomised controlled trials and observational studies, including case series and cohort studies, and relevant meta-analyses.

Information sources

Literature search strategies were developed using medical subject headings (MeSH) and text words related to CPAP, pneumonia and lower respiratory tract infection, and children. We searched MEDLINE (OVID interface), EMBASE (OVID interface) and PubMed for all relevant published papers.

We scanned the reference lists of included studies or relevant reviews identified through the search.

Where appropriate, authors were contacted to get more information on the contexts in which their studies took place.

Search strategy

Both qualitative and quantitative studies were sought. No study design, date or language limits were imposed on the search. Medline, EMBASE, and PubMed were searched from inception. The specific search strategies were created by a Health Sciences Librarian with expertise in systematic review searching. The MEDLINE strategy was developed with input from the project team. The draft MEDLINE search strategy is included below. This was then adapted to the syntax and subject headings of the other databases.

Study Records

Data management

All search results were saved in a shared file accessible to all members of the study team, and citations saved in EndNote. When reviewing papers for inclusion and exclusion records accessible to all team members were kept in the shared folder and on password protected computers.

Selection process

The titles and abstracts of all papers identified though the search were reviewed by two independent reviewers for inclusion or exclusion according to the above criteria. We obtained full reports for all titles that appeared to meet the inclusion criteria or where there was any uncertainty.

Two independent reviewers screened the full text reports and decided whether these meet the inclusion criteria. We sought additional information from study authors where necessary to resolve questions about eligibility. We resolved disagreements through discussion with the team. We recorded the reasons for excluding trials. Neither of the review authors were blind to the journal titles or to the study authors or institutions.

Data collection process

We used a standardised form of specific items (see below) with which 2 independent reviewers extracted data from each eligible study and entered in to an Excel spreadsheet. Reviewers resolved disagreements by discussion, and any unresolved disagreements were discussed with the whole team. We contacted study authors to resolve any uncertainties.

Data Items

For all papers included in the study we sought the following information regarding potential variables and context in which the study took place. We documented whether this data was available in the published papers or whether it was acquired from contact with the authors or other means. If we were unable to find this data this was also noted.

General:

- Type of publication (peer-reviewed journal, non-academic, agency report, etc.)
- Type of research (Randomised controlled trial, cohort, case-series etc).
- Year of publication
- Year of study (date range)
- Geographic location(s) - WHO region, country, sub-national region
- World bank income category (LIC, LMIC, UMIC)

Population:

- Participant number (total and disaggregated by age)
    - Age (2-11 months, 1-4 years)
    - Sex
- Prevalence of comorbidities (malnutrition, HIV, TB, chronic respiratory illnesses, etc.)
- Comparison population, if reported
- Inclusion criteria
- Exclusion criteria

CPAP equipment:

- Ventilator, bubble CPAP, CPAP driver, brand, commercial or otherwise
- Gas flow source
- Oxygen sources
- Blender mechanism
- Circuit tubing diameter and composition

- Bottle used for insertion of expiratory limb
- Nasal interface
- Humidification method
- Commercial or home-made
- Cost

CPAP clinical method

- Gas/oxygen flow rates used
- Fraction of inspired oxygen (starting, maximum)

Monitoring and supportive care

- Pulse oximetry
- Paediatric monitoring charts with defined responses and escalation procedures
- Respiratory Distress Score
- Fluids and feeding
- Guidelines for the management of pneumonia and other causes of respiratory distress
- Guidelines for the use of all CPAP and oxygen equipment

Health service aspects

- Urban or rural
- Tertiary, provincial/state, or district hospital
- Separate PICU, general children's ward or intensive care area in children's ward
- Nurse-patient ratio, day and night
- Frequency of clinical review
- Paediatrician presence
- Other doctor presence
- Nurses with training in CPAP
- Nurses with specific training in paediatric intensive care
- Chest x-ray facilities
- Frequency of ward rounds

CPAP maintenance

- Biomedical technician trained to repair CPAP machines and other oxygen equipment
- Oxygen analyser for checking performance of oxygen equipment

Outcomes

For all papers included in the study we sought the following information regarding outcomes. The primary outcomes reviewed were:

- Mortality rates
  - Overall
  - Age specific (28 days - 11 months, 1-5 years)
  - Pneumonia
  - Bronchiolitis
  - With HIV

- With cardiac disease
  - With neurodevelopmental disease
  - With malnutrition
- With chronic respiratory disease
- With other pre-existing co-morbidity

- Adverse events
  - Pneumothorax
  - Nasal bleeding or pressure areas
  - Other

Secondary outcomes that we reviewed were:

- Length of Stay
- Change in clinical signs
  - Respiratory rate
  - Oxygen saturations
  - Heart rate
- Treatment failure rates
- Rates of children needing intubation
- Other issues raised regarding
  - acceptability
  - feasibility
  - unintended effects
  - practice points
  - required capacity

Quality Assessment and Risk of Bias in individual studies

Included studies may or may not have a comparison group. To assess the quality of and risk of bias within included studies, the methodological quality of potential studies was assessed by using the Effective Public Healthcare Panacea Project (EPHPP) QA Tool. Using this tool, studies were rated as strong, moderate or weak with respect to selection bias, study design, confounders, blinding, data collection method, withdrawals and dropouts, and a global rating. This was undertaken by two separate reviewers. Where there was disagreement, a third reviewer was used as an arbitrator.

Appendix S2 – MEDLINE database search

Database: Ovid MEDLINE(R) ALL <1946 to August 19, 2020>

Search Strategy:

----

1 exp *Pneumonia/ (82613)

2 ((respiratory adj3 (infection* or distress or failure or disease* or illness*)) or pneumonia or pneumonias or lung-inflammation* or lobitis or nonspecific-inflammatory-lung-disease* or peripneumonia or pleuropneumonia or pleuropneumonitis or pneumonic-lung* or pneumonic-pleurisy or pneumonic-pleuritis or pneumonitides or pneumonitis or pulmonal-inflammation* or pulmonary-inflammation* or pulmonic-inflammation* or bronchiolitis).tw,kf. (296333)

3 *Pneumococcal Infections/ (10117)

4 exp *Bronchiolitis/ (6581)

5 *respiration disorders/th or *respiratory distress syndrome, adult/th or exp *respiratory distress syndrome, newborn/th (7338)

6 1 or 2 or 3 or 4 or 5 (335894)

7 *continuous positive airway pressure/ (4610)

8 (continuous-positive-airway-pressure or cpap or ncpap or bcpap or positive-end-expiratory-pressure or peep).tw,kf. (20303)

9 7 or 8 (21172)

10 (infan* or toddler* or pre-schooler* or preschooler* or kinder or kinders or kindergarten* or kinder-aged or boy or boys or girl or girls or child or children or childhood or youngster* or kid or kids or pediatric* or paediatric*).af. (3632966)

11 developing countries/ (74913)

12 (austere or (limited adj2 resource*) or (low adj2 resource*) or (transitioning adj econom*) or (third adj world) or LMIC or LMICs or (lami adj countr*) or (transitional adj countr*) or (low adj gdp) or (low adj gnp) or (low adj gross adj domestic) or (low adj gross adj national) or ((emerging or developing or (low adj income) or (middle adj income) or (low adj3 middle) or underdeveloped or under-developed or (less* adj developed) or underserved or under-served or deprived or poor*) and (countr* or nation*1 or econom* or population or world))).tw,kf. (387339)

13 exp africa/ (266978)

14 americas/ or exp caribbean region/ or exp central america/ or latin america/ or mexico/ or exp south america/ (253103)

15 europe/ or exp europe, eastern/ or exp transcaucasia/ (283689)

16 antarctic regions/ or exp atlantic islands/ or exp indian ocean islands/ or exp pacific islands/ (78754)

17 New Guinea/ (2062)

18 asia/ or exp asia, central/ or asia, southeastern/ or borneo/ or cambodia/ or east timor/ or indonesia/ or laos/ or malaysia/ or mekong valley/ or myanmar/ or philippines/ or thailand/ or vietnam/ or asia, western/ or bangladesh/ or bhutan/ or india/ or middle east/ or afghanistan/ or iran/ or iraq/ or jordan/ or lebanon/ or oman/ or saudi arabia/ or syria/ or turkey/ or yemen/ or nepal/ or pakistan/ or sri lanka/ or far east/ or china/ or tibet/ or exp korea/ or mongolia/ (569980)

19 (Afghanistan or Albania or Algeria or Angola or Antigua or Argentina or Armenia* or Aruba or Azerbaijan or Bahrain or Bangladesh or Barbados or Barbuda or Belarus or Byelarus* or Byelorussian or Belorussian or Belorus* or Belize or Benin or Bhutan or Bolivia or Bosnia or Botswana or Brasil or Brazil or Bulgaria or (Burkina adj Fas*) or (Upper adj Volta) or Burma or Burundi or Cambodia or Khmer or Kampuchea or Cameron* or Cameroon* or (Cape adj Verde) or (Cabo adj Verde) or (Central adj African adj Republic) or Chad or Chile or China or Colombia or Comoros or (Comoro adj Island*) or Comores or Mayotte or Congo or Kongo or (Cook adj Island*) or (Costa adj Rica) or (Cote adj D'ivoire) or Croatia or Cuba or Cyprus or Czech* or Djibouti or Dominica or Dominican or (East adj Timor) or (East adj Timur) or Ecuador or Egypt or El-Salvador or (Equatorial adj Guinea) or Eritrea or Estonia or Ethiopia or Fiji or (French adj Somaliland) or Futuna or Gabon or (Gabonese adj

Republic)
or Gambia or Gaza or (Georgia* adj Republic) or Ghana or Grenada or Guam or Guatemala or Guinea or Guiana or Guyana or Haiti or Herzeg* or Hercegovina or Honduras or Hungary or India or Indonesia or Iran or Iraq or (Ivory adj Coast) or Jamaica or Jordan or Kazakh* or Kenya or Kiribati or Korea or Kosovo or (Kyrgyz adj Republic) or Kyrgyzstan or Kirghizia or Kirghiz or Kirgizstan or Laos or (Lao* adj2 Democratic adj Republic) or (Lao* adj PDR) or Latvia or Lebanon or Lesotho or Basutoland or Liberia or Libya or Lithuania or Macedonia or Madagascar or (Magalasy adj Republic) or Malawi or Malay* or Sabah or Sarawak or Maldives or Mali or (Marshall adj Island*) or Mauritania or Mauritius or (Agalega adj Island*) or Mexico or Micronesia or Moldov* or Mongolia or Montserrat or Montenegro or Morocco or Ifni or Mozambique or Myanma* or Namibia or Nauru or Nepal or (Netherlands adj Antilles) or (Dutch adj Antilles) or (New adj Guinea) or (New adj Caledonia) or Nicaragua or Niue or Niger or Nigeria or (Northern adj Mariana adj Island*) or Nyasaland or Oman or Pakistan or Palau or Panama or (Papua adj New adj Guinea) or PNG or Palestine or Paraguay or Peru or Philipines or Philippines or Phillipines or Phillippines or Poland or (Puerto adj Rico) or Yemen or Romania or Roumania or Rumania or Russia* or Rwanda or Ruanda or (Saint adj Kitts) or (St adj Kitts) or Nevis or (Saint adj Vincent) or (St adj Vincent) or Grenadines or Samoa* or (Navigator adj Island*) or (Saint adj Lucia) or (St adj Lucia) or (Saint adj Helena) or (St adj Helena) or (Sao adj Tome) or (Saudi adj Arabia) or Senegal or Serbia or Seychelles or (Sierra adj Leone) or Slovenia or Slovak* or (South adj Africa) or (Solomon adj Island*) or Somalia or (Sri adj Lanka) or Ceylon or Sudan or Surinam* or Swaziland or Syria or Tajikistan or Tadzhikistan or Tadjikistan or Tadzhik or Tanzania or Thailand or Tibet or Timor-Leste or Togo or (Togolese adj Republic) or Tokelau or Tonga or Trinidad or Tobago or Tunisia or Turkey or Turkmenistan or Turkmen or Tuval!
u or Uganda or Ukraine or Uruguay or Urundi or USSR or (Soviet adj Union) or "Union of Soviet Socialist Republics" or Uzbekistan or Vanuatu or (New adj Hebrides) or Venezuela or Vietnam or (Viet adj Nam) or (Wallis adj2 Futuna) or (United adj Arab adj Republic) or (West adj Bank) or (West adj Indies) or Yemen or Yugoslavia or Zaire or Zambia or Zimbabwe or Rhodesia).tw,kf. (1269881)

20 (africa or americas or caribbean or (central adj America) or (latin adj America) or (south adj America) or (eastern adj Europe) or Transcaucasia or antarctic or (atlantic adj island*) or (indian adj ocean adj island*) or (pacific adj island*) or polynesia or (central adj asia) or (southeast* adj asia) or (south-east* adj asia) or borneo or mekong or (western adj asia) or (middle adj east) or (far adj east)).tw,kf. (231060)

21 11 or 12 or 13 or 14 or 15 or 16 or 17 or 18 or 19 or 20 (2245593)

22 6 and 9 and 10 and 21 (244)

Table S1. Inclusion and exclusion criteria for studies in this review

Inclusion criteria	*Exclusion criteria*
1. Observational or interventional study or meta-analysis involving original data or analysis<br>2. Published in the year 2000 or later<br>3. Published in English<br>4. Includes children aged between 28 days and 5 years of age and it is possible to extract data specifically relating to children within these age groups from the data available.<br>5. Included children whose primary presentation is pneumonia, as defined by WHO criteria, which may include other types of acute respiratory infection such as bronchiolitis<br>6. One or more children in the study, with a primary presentation of pneumonia received CPAP therapy, and it is possible to extract data specifically relating to those children who received CPAP.<br>7. Wholly or partially undertaken in LMICs, as defined by World Bank	1. Does not provide original data (e.g. review articles, editorials)<br>2. Conducted in a neonatal unit/neonatal ICU, or focuses only on neonates below 40 weeks' gestation

Table S2. EPHPP quality assessment of included studies

Paper	Selection Bias	Study design	Confounders	Blinding	Data collection methods	Withdrawal and drop-outs	Overall
Anitha 2016	weak	weak	weak	weak	weak	strong	weak
Bjorkland 2018	moderate	weak	weak	weak	weak	strong	weak
Cesar 2018	moderate	strong	not reported	weak	not reported	not reported	weak
Chisti 2015	strong	moderate	moderate	weak	strong	strong	moderate
Chisti 2018	moderate	moderate	moderate	weak	moderate	not reported	moderate
Jayashree 2016	moderate	moderate	moderate	weak	moderate	moderate	moderate
Kinikar 2011	weak	moderate	weak	weak	moderate	moderate	weak
Lal 2018	weak	strong	moderate	weak	weak	strong	moderate
Machen 2015	moderate	weak	moderate	weak	moderate	strong	moderate
McCollum 2019	moderate	strong	strong	weak	moderate	strong	strong
Myers 2019	moderate	weak	moderate	weak	moderate	strong	moderate
Pulsan 2019	strong	weak	moderate	weak	moderate	strong	moderate
Sarkar 2018	mod	strong	moderate	weak	moderate	strong	moderate
Walk 2016	moderate	moderate	moderate	weak	moderate	strong	moderate
Wilson 2013	moderate	strong	moderate	weak	moderate	strong	moderate
Wilson 2017	strong	strong	moderate	weak	moderate	strong	strong
Brown 2013*	weak	Weak	weak	weak	moderate	strong	weak
Larsen 2020*	weak	Weak	weak	weak	weak	n/a	weak
McCollum 2011*	weak	Weak	weak	weak	moderate	n/a	weak

Non-clinical studies not included in quality assessment as they do not present clinical outcome data.
*Case reports
